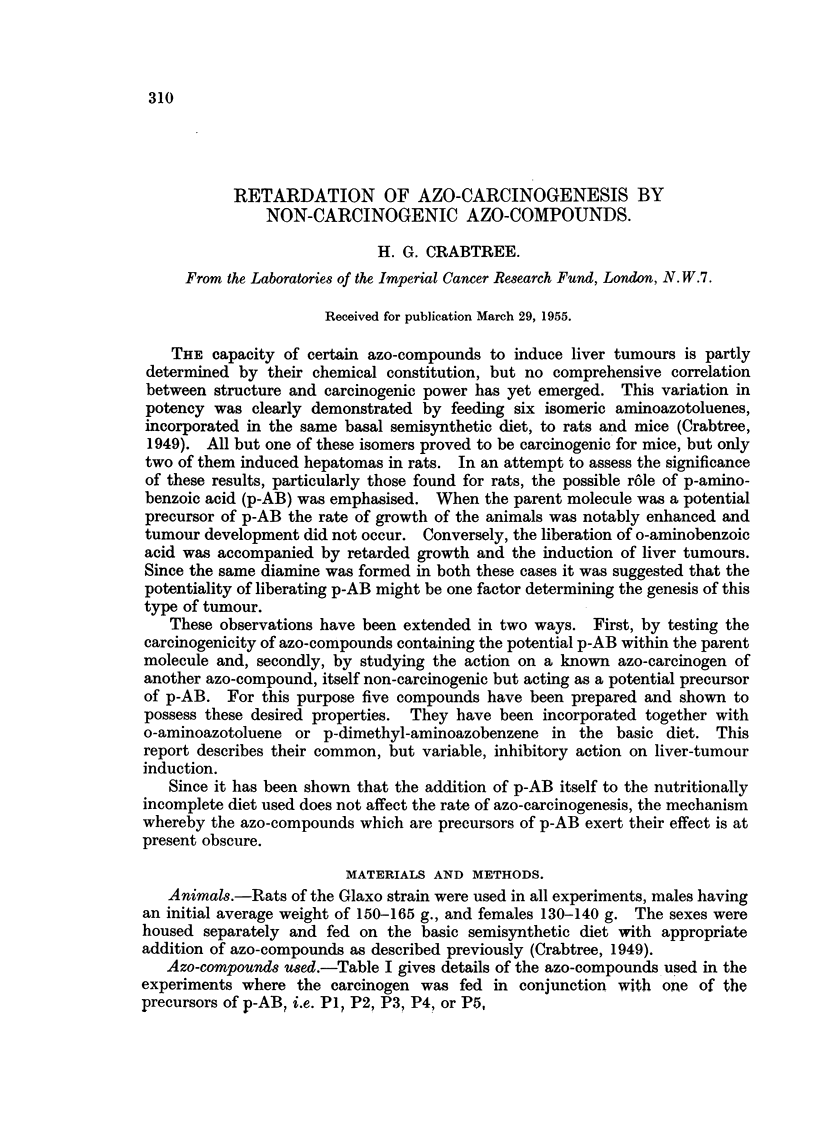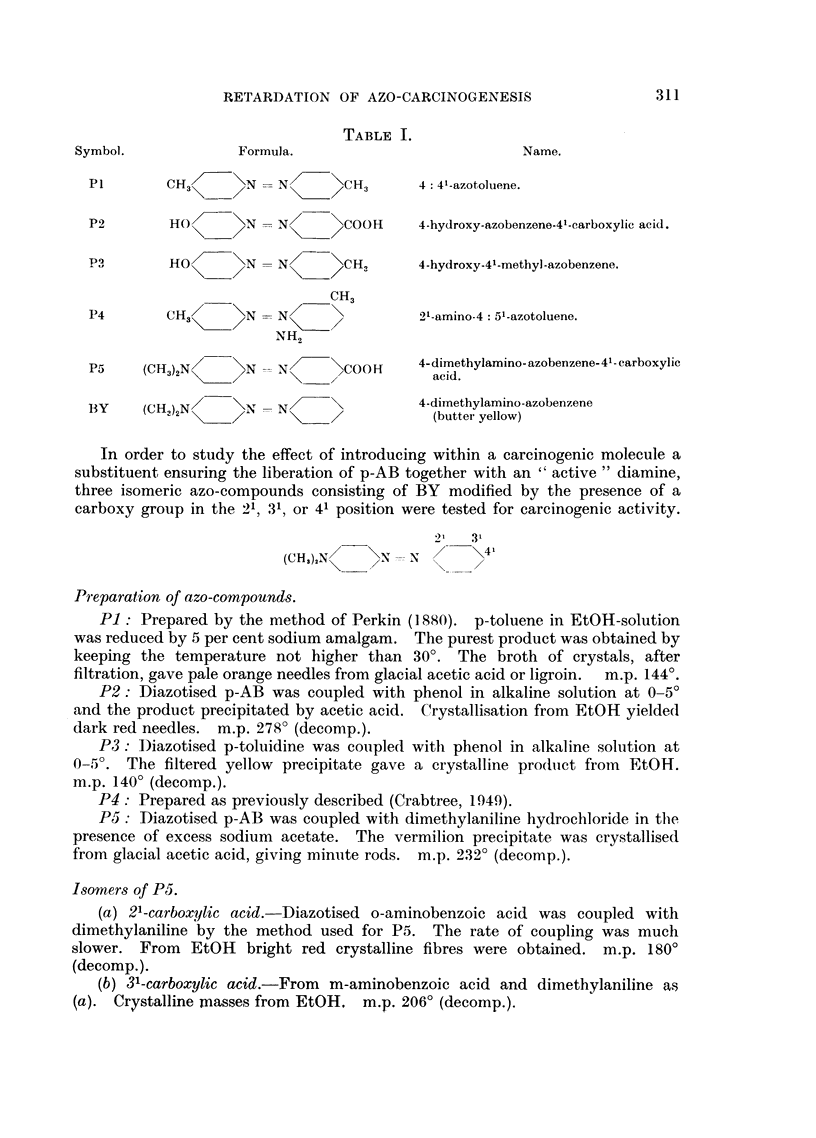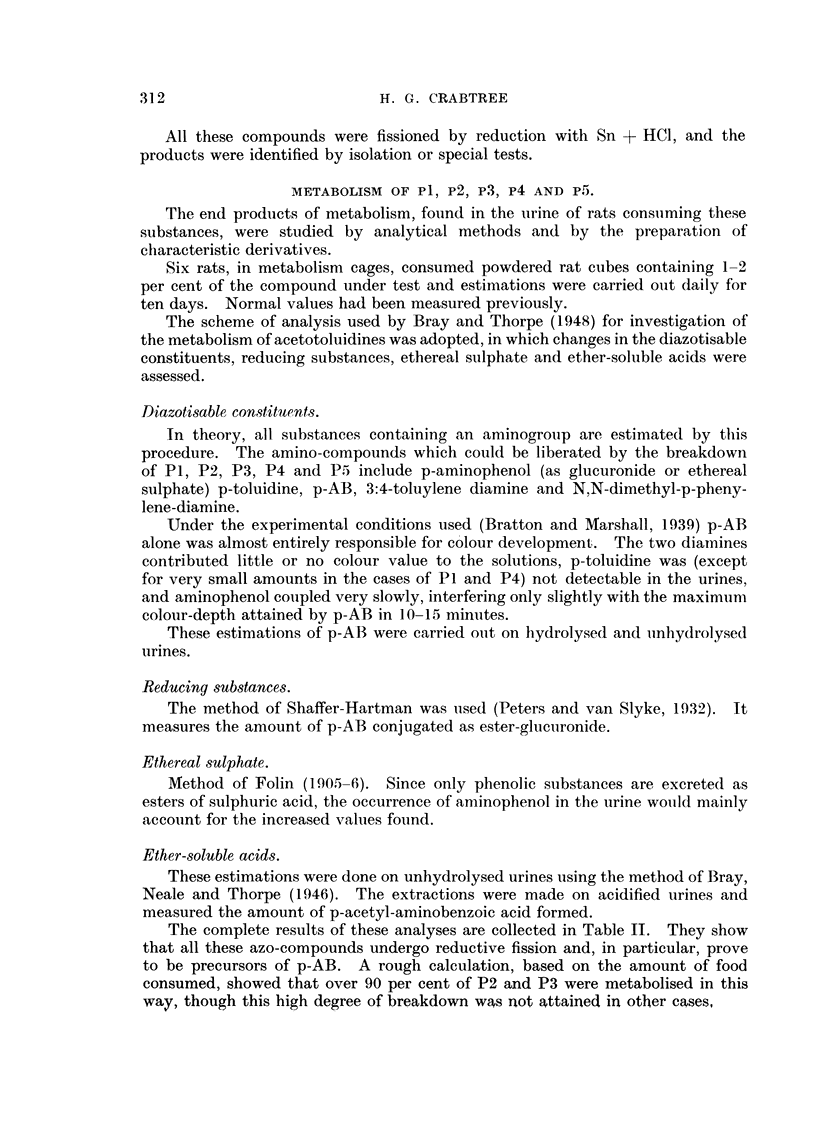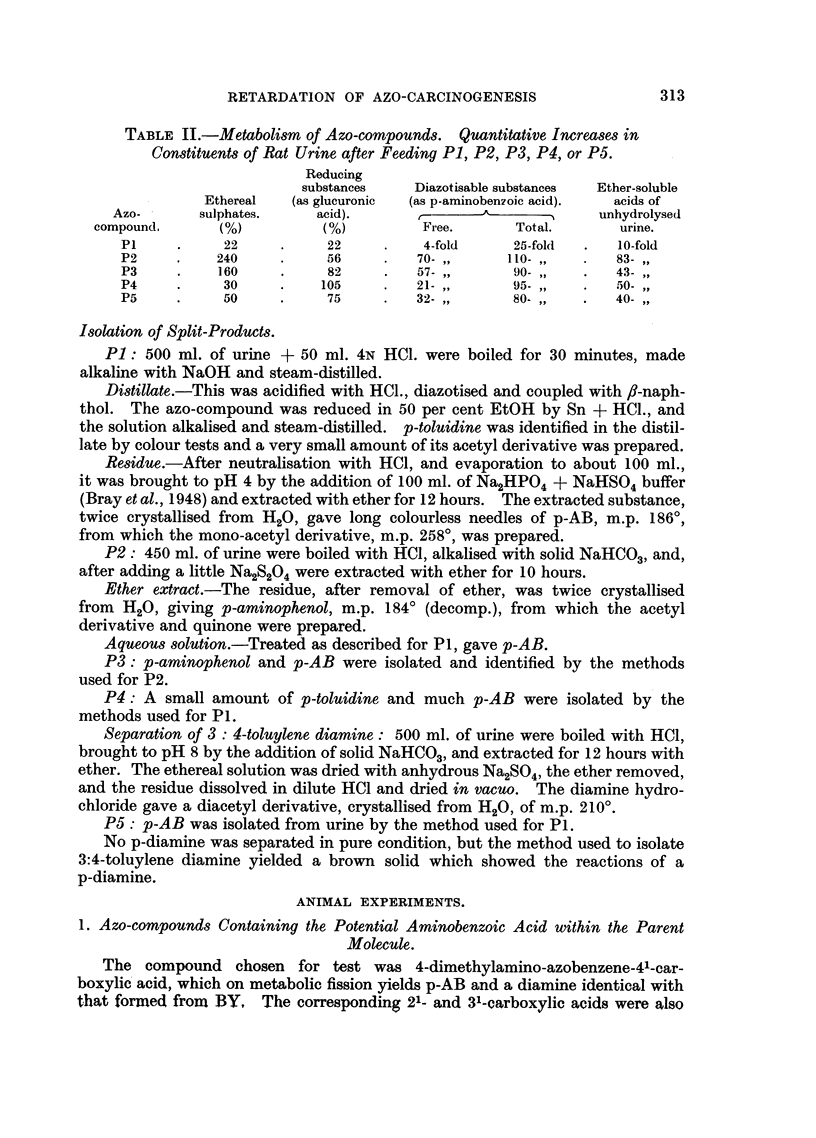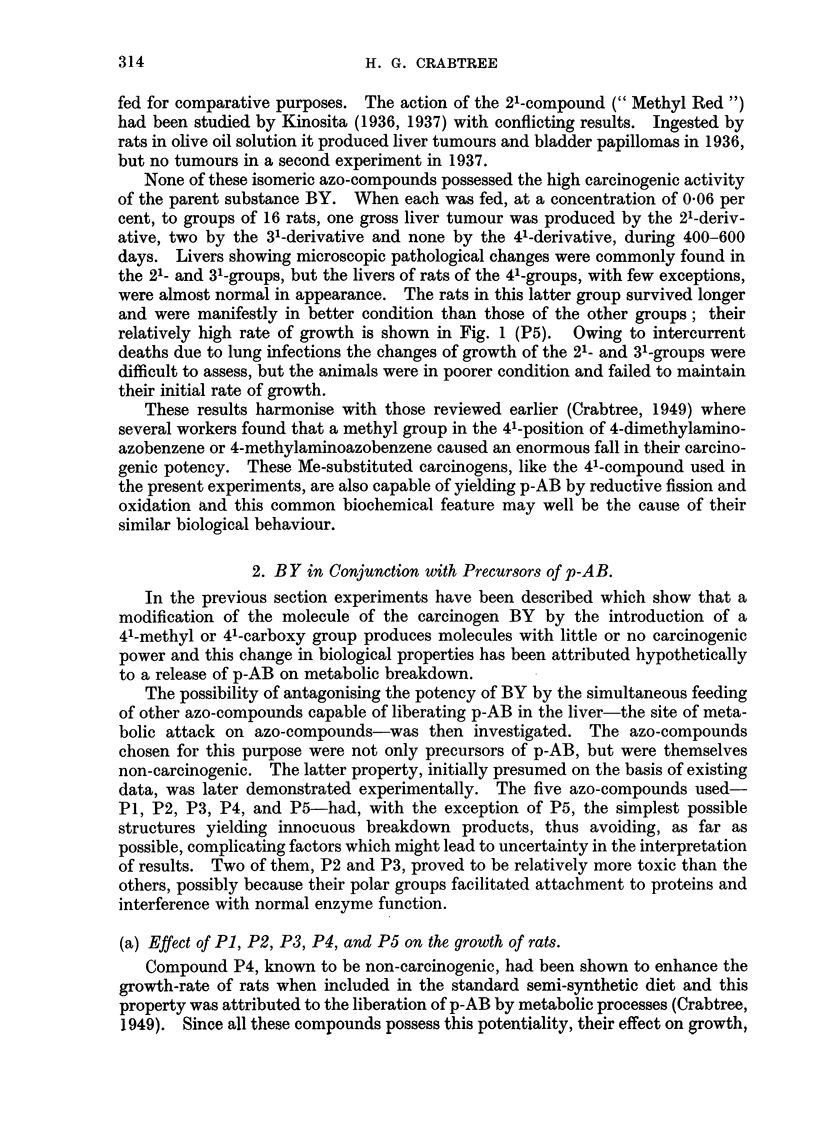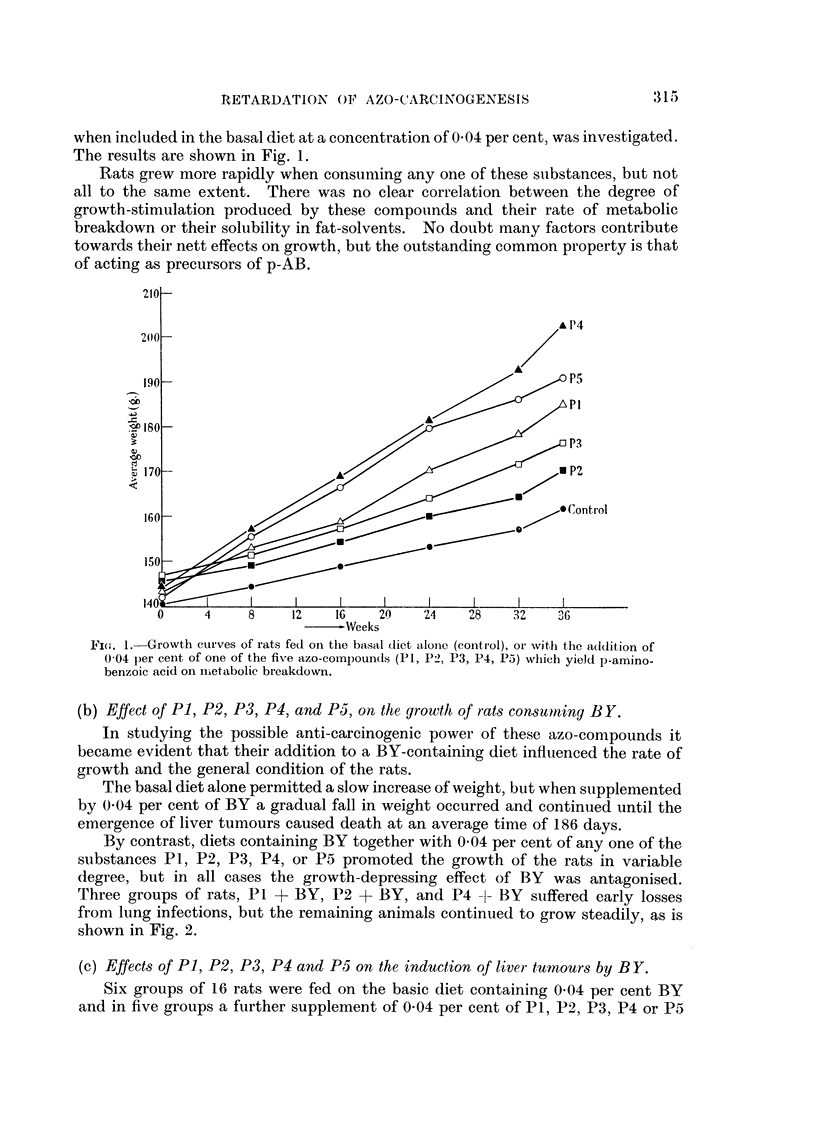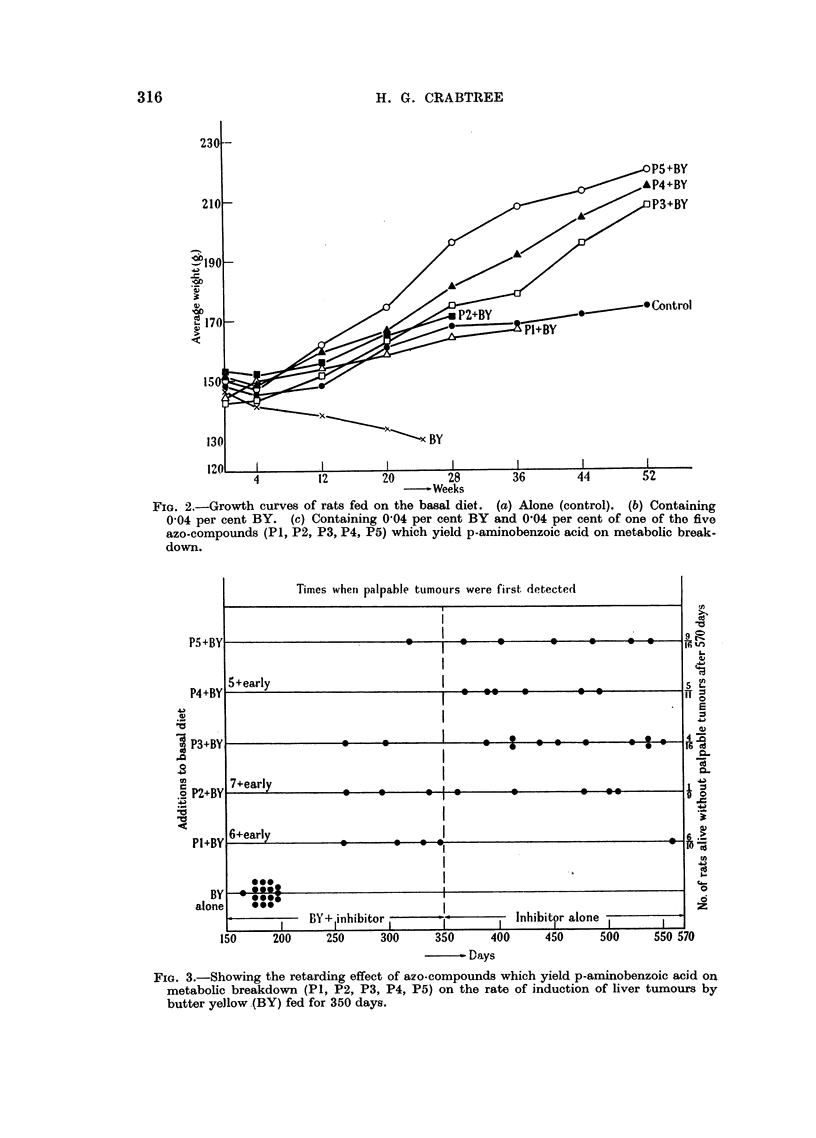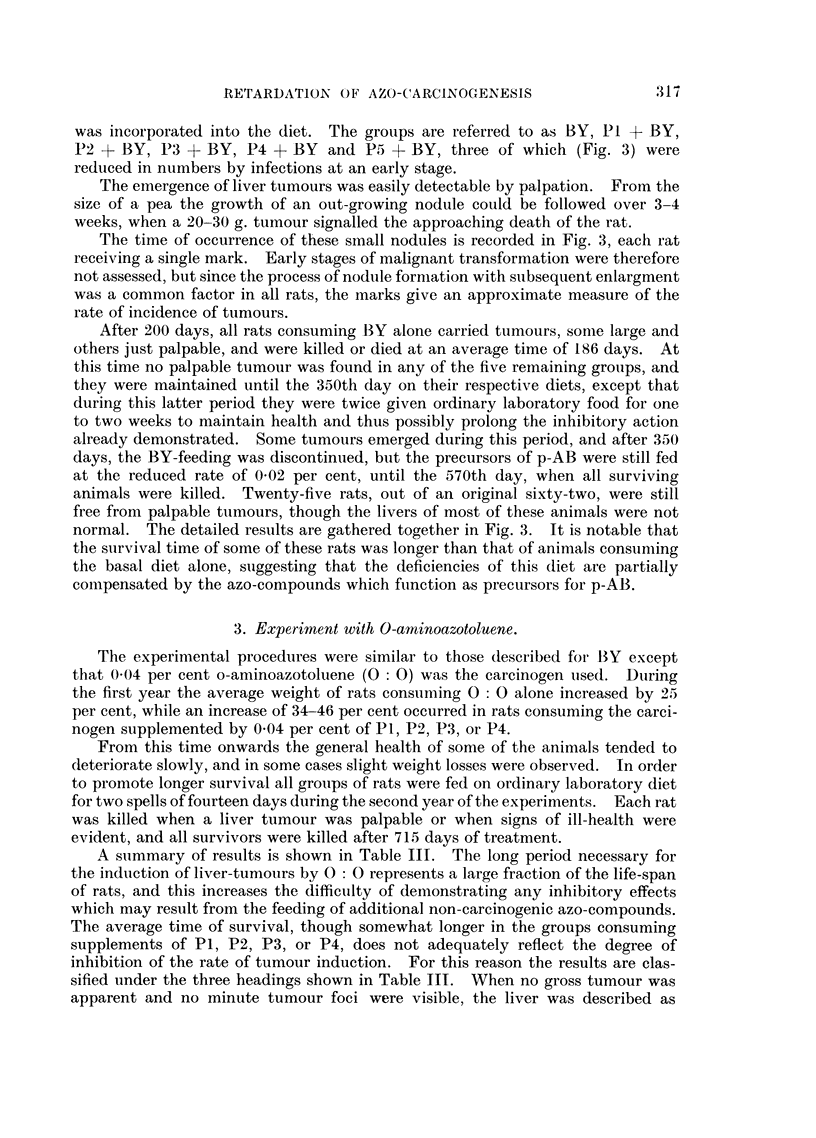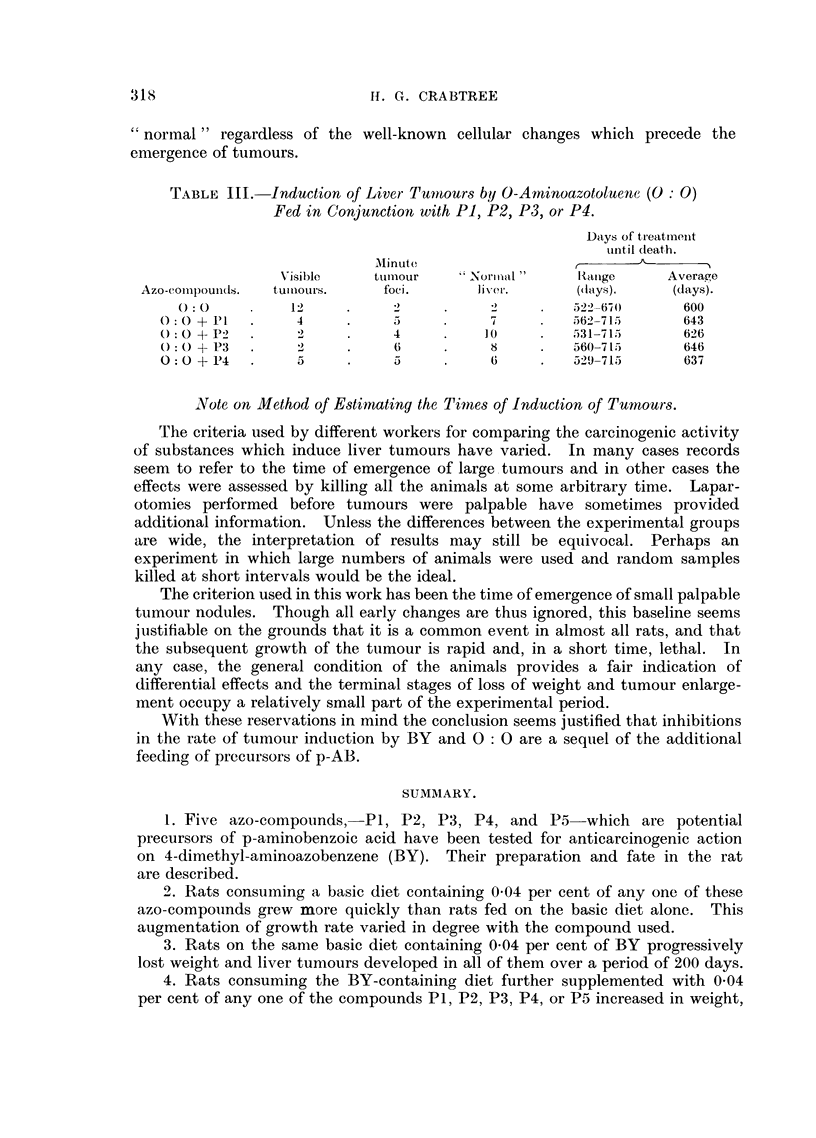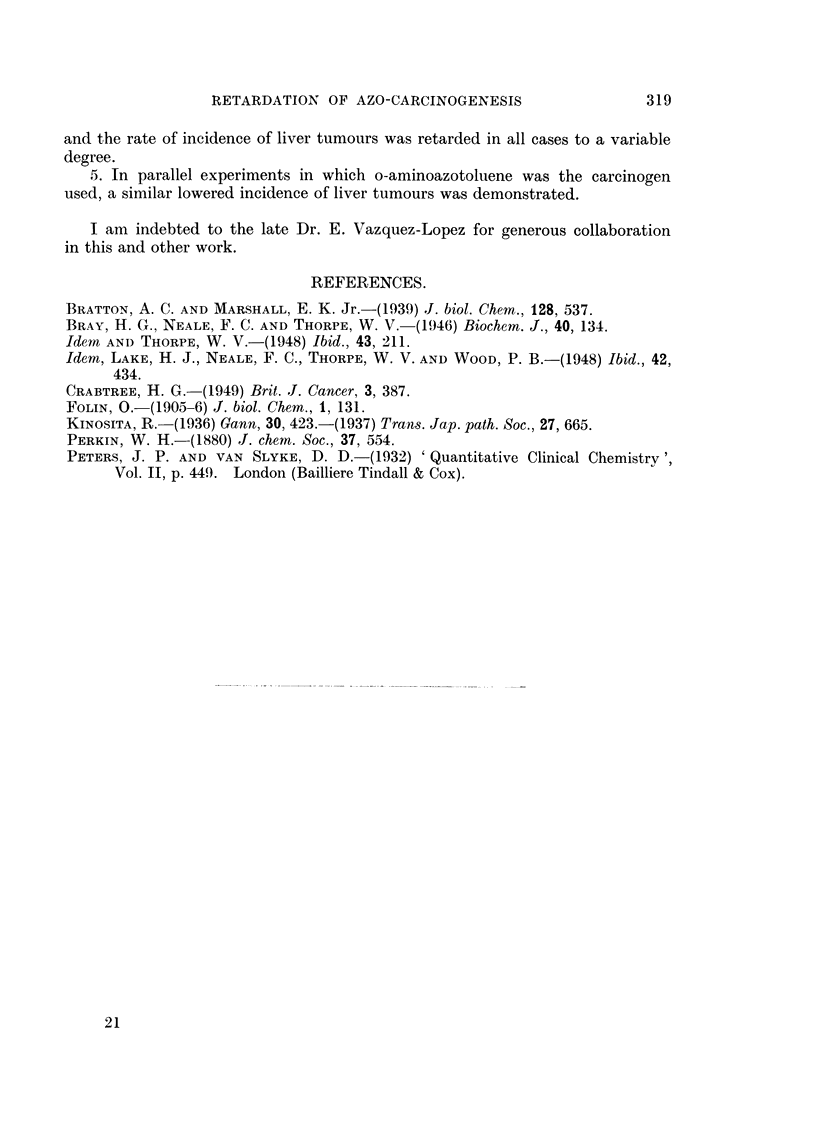# Retardation of Azo-Carcinogenesis by Non-Carcinogenic Azo-Compounds

**DOI:** 10.1038/bjc.1955.28

**Published:** 1955-06

**Authors:** H. G. Crabtree


### RETARDATION OF AZO-CARCINOGENESIS BY NON-CARCINOGENIC AZO-COMPOUNDS.

H. G. CRABTREE.

*From the Laboratories of the Imperial Cancer Research Fund, London, N.W.7.*

Received for publication March 29, 1955.

THE capacity of certain azo-compounds to induce liver tumours is partly determined by their chemical constitution, but no comprehensive correlation between structure and carcinogenic power has yet emerged. This variation in potency was clearly demonstrated by feeding six isomeric aminoazotoluenes, incorporated in the same basal semisynthetic diet, to rats and mice (Crabtree, 1949). All but one of these isomers proved to be carcinogenic for mice, but only two of them induced hepatomas in rats. In an attempt to assess the significance of these results, particularly those found for rats, the possible rôle of p-amino-benzoic acid (p-AB) was emphasised. When the parent molecule was a potential precursor of p-AB the rate of growth of the animals was notably enhanced and tumour development did not occur. Conversely, the liberation of o-aminobenzoic acid was accompanied by retarded growth and the induction of liver tumours. Since the same diamine was formed in both these cases it was suggested that the potentiality of liberating p-AB might be one factor determining the genesis of this type of tumour.

These observations have been extended in two ways. First, by testing the carcinogenicity of azo-compounds containing the potential p-AB within the parent molecule and, secondly, by studying the action on a known azo-carcinogen of another azo-compound, itself non-carcinogenic but acting as a potential precursor of p-AB. For this purpose five compounds have been prepared and shown to possess these desired properties. They have been incorporated together with o-aminoazotoluene or p-dimethyl-aminoazobenzene in the basic diet. This report describes their common, but variable, inhibitory action on liver-tumour induction.

Since it has been shown that the addition of p-AB itself to the nutritionally incomplete diet used does not affect the rate of azo-carcinogenesis, the mechanism whereby the azo-compounds which are precursors of p-AB exert their effect is at present obscure.

### MATERIALS AND METHODS.

*Animals.*—Rats of the Glaxo strain were used in all experiments, males having an initial average weight of 150–165 g., and females 130–140 g. The sexes were housed separately and fed on the basic semisynthetic diet with appropriate addition of azo-compounds as described previously (Crabtree, 1949).

*Azo-compounds used.*—Table I gives details of the azo-compounds used in the experiments where the carcinogen was fed in conjunction with one of the precursors of p-AB, *i.e.* P1, P2, P3, P4, or P5,

TABLE I.

| Symbol. | Formula. | Name. |
|---|---|---|
| P1 |  | 4 : $4^1$-azotoluene. |
| P2 |  | 4-hydroxy-azobenzene-$4^1$-carboxylic acid. |
| P3 |  | 4-hydroxy-$4^1$-methyl-azobenzene. |
| P4 |  | $2^1$-amino-4 : $5^1$-azotoluene. |
| P5 |  | 4-dimethylamino-azobenzene-$4^1$-carboxylic acid. |
| BY |  | 4-dimethylamino-azobenzene (butter yellow) |

In order to study the effect of introducing within a carcinogenic molecule a substituent ensuring the liberation of p-AB together with an " active " diamine, three isomeric azo-compounds consisting of BY modified by the presence of a carboxy group in the $2^1$, $3^1$, or $4^1$ position were tested for carcinogenic activity.

*Preparation of azo-compounds.*

*P1 :* Prepared by the method of Perkin (1880). p-toluene in EtOH-solution was reduced by 5 per cent sodium amalgam. The purest product was obtained by keeping the temperature not higher than 30°. The broth of crystals, after filtration, gave pale orange needles from glacial acetic acid or ligroin. m.p. 144°.

*P2 :* Diazotised p-AB was coupled with phenol in alkaline solution at 0–5° and the product precipitated by acetic acid. Crystallisation from EtOH yielded dark red needles. m.p. 278° (decomp.).

*P3 :* Diazotised p-toluidine was coupled with phenol in alkaline solution at 0–5°. The filtered yellow precipitate gave a crystalline product from EtOH. m.p. 140° (decomp.).

*P4 :* Prepared as previously described (Crabtree, 1949).

*P5 :* Diazotised p-AB was coupled with dimethylaniline hydrochloride in the presence of excess sodium acetate. The vermilion precipitate was crystallised from glacial acetic acid, giving minute rods. m.p. 232° (decomp.).

*Isomers of P5.*

(*a*) *$2^1$-carboxylic acid.*—Diazotised o-aminobenzoic acid was coupled with dimethylaniline by the method used for P5. The rate of coupling was much slower. From EtOH bright red crystalline fibres were obtained. m.p. 180° (decomp.).

(*b*) *$3^1$-carboxylic acid.*—From m-aminobenzoic acid and dimethylaniline as (*a*). Crystalline masses from EtOH. m.p. 206° (decomp.).

All these compounds were fissioned by reduction with Sn + HCl, and the products were identified by isolation or special tests.

### METABOLISM OF P1, P2, P3, P4 AND P5.

The end products of metabolism, found in the urine of rats consuming these substances, were studied by analytical methods and by the preparation of characteristic derivatives.

Six rats, in metabolism cages, consumed powdered rat cubes containing 1–2 per cent of the compound under test and estimations were carried out daily for ten days. Normal values had been measured previously.

The scheme of analysis used by Bray and Thorpe (1948) for investigation of the metabolism of acetotoluidines was adopted, in which changes in the diazotisable constituents, reducing substances, ethereal sulphate and ether-soluble acids were assessed.

*Diazotisable constituents.*

In theory, all substances containing an aminogroup are estimated by this procedure. The amino-compounds which could be liberated by the breakdown of P1, P2, P3, P4 and P5 include p-aminophenol (as glucuronide or ethereal sulphate) p-toluidine, p-AB, 3:4-toluylene diamine and N,N-dimethyl-p-phenylene-diamine.

Under the experimental conditions used (Bratton and Marshall, 1939) p-AB alone was almost entirely responsible for colour development. The two diamines contributed little or no colour value to the solutions, p-toluidine was (except for very small amounts in the cases of P1 and P4) not detectable in the urines, and aminophenol coupled very slowly, interfering only slightly with the maximum colour-depth attained by p-AB in 10–15 minutes.

These estimations of p-AB were carried out on hydrolysed and unhydrolysed urines.

*Reducing substances.*

The method of Shaffer-Hartman was used (Peters and van Slyke, 1932). It measures the amount of p-AB conjugated as ester-glucuronide.

*Ethereal sulphate.*

Method of Folin (1905–6). Since only phenolic substances are excreted as esters of sulphuric acid, the occurrence of aminophenol in the urine would mainly account for the increased values found.

*Ether-soluble acids.*

These estimations were done on unhydrolysed urines using the method of Bray, Neale and Thorpe (1946). The extractions were made on acidified urines and measured the amount of p-acetyl-aminobenzoic acid formed.

The complete results of these analyses are collected in Table II. They show that all these azo-compounds undergo reductive fission and, in particular, prove to be precursors of p-AB. A rough calculation, based on the amount of food consumed, showed that over 90 per cent of P2 and P3 were metabolised in this way, though this high degree of breakdown was not attained in other cases.

TABLE II.—*Metabolism of Azo-compounds. Quantitative Increases in Constituents of Rat Urine after Feeding P1, P2, P3, P4, or P5.*

| Azo-compound. | Ethereal sulphates. (%) | Reducing substances (as glucuronic acid). (%) | Diazotisable substances (as p-aminobenzoic acid). Free. | Total. | Ether-soluble acids of unhydrolysed urine. |
|---|---|---|---|---|---|
| P1 | 22 | 22 | 4-fold | 25-fold | 10-fold |
| P2 | 240 | 56 | 70- „ | 110- „ | 83- „ |
| P3 | 160 | 82 | 57- „ | 90- „ | 43- „ |
| P4 | 30 | 105 | 21- „ | 95- „ | 50- „ |
| P5 | 50 | 75 | 32- „ | 80- „ | 40- „ |

*Isolation of Split-Products.*

*P1 :* 500 ml. of urine + 50 ml. 4N HCl. were boiled for 30 minutes, made alkaline with NaOH and steam-distilled.

*Distillate.*—This was acidified with HCl., diazotised and coupled with $\beta$-naphthol. The azo-compound was reduced in 50 per cent EtOH by Sn + HCl., and the solution alkalised and steam-distilled. *p-toluidine* was identified in the distillate by colour tests and a very small amount of its acetyl derivative was prepared.

*Residue.*—After neutralisation with HCl, and evaporation to about 100 ml., it was brought to pH 4 by the addition of 100 ml. of $Na_2HPO_4$ + $NaHSO_4$ buffer (Bray *et al.*, 1948) and extracted with ether for 12 hours. The extracted substance, twice crystallised from $H_2O$, gave long colourless needles of p-AB, m.p. 186°, from which the mono-acetyl derivative, m.p. 258°, was prepared.

*P2 :* 450 ml. of urine were boiled with HCl, alkalised with solid $NaHCO_3$, and, after adding a little $Na_2S_2O_4$ were extracted with ether for 10 hours.

*Ether extract.*—The residue, after removal of ether, was twice crystallised from $H_2O$, giving *p-aminophenol*, m.p. 184° (decomp.), from which the acetyl derivative and quinone were prepared.

*Aqueous solution.*—Treated as described for P1, gave *p-AB.*

*P3 : p-aminophenol* and *p-AB* were isolated and identified by the methods used for P2.

*P4 :* A small amount of *p-toluidine* and much *p-AB* were isolated by the methods used for P1.

*Separation of 3 : 4-toluylene diamine :* 500 ml. of urine were boiled with HCl, brought to pH 8 by the addition of solid $NaHCO_3$, and extracted for 12 hours with ether. The ethereal solution was dried with anhydrous $Na_2SO_4$, the ether removed, and the residue dissolved in dilute HCl and dried *in vacuo.* The diamine hydrochloride gave a diacetyl derivative, crystallised from $H_2O$, of m.p. 210°.

*P5 : p-AB* was isolated from urine by the method used for P1.

No p-diamine was separated in pure condition, but the method used to isolate 3:4-toluylene diamine yielded a brown solid which showed the reactions of a p-diamine.

### ANIMAL EXPERIMENTS.

### 1. *Azo-compounds Containing the Potential Aminobenzoic Acid within the Parent Molecule.*

The compound chosen for test was 4-dimethylamino-azobenzene-$4^1$-carboxylic acid, which on metabolic fission yields p-AB and a diamine identical with that formed from BY. The corresponding $2^1$- and $3^1$-carboxylic acids were also fed for comparative purposes. The action of the $2^1$-compound (" Methyl Red ") had been studied by Kinosita (1936, 1937) with conflicting results. Ingested by rats in olive oil solution it produced liver tumours and bladder papillomas in 1936, but no tumours in a second experiment in 1937.

None of these isomeric azo-compounds possessed the high carcinogenic activity of the parent substance BY. When each was fed, at a concentration of 0·06 per cent, to groups of 16 rats, one gross liver tumour was produced by the $2^1$-derivative, two by the $3^1$-derivative and none by the $4^1$-derivative, during 400–600 days. Livers showing microscopic pathological changes were commonly found in the $2^1$- and $3^1$-groups, but the livers of rats of the $4^1$-groups, with few exceptions, were almost normal in appearance. The rats in this latter group survived longer and were manifestly in better condition than those of the other groups ; their relatively high rate of growth is shown in Fig. 1 (P5). Owing to intercurrent deaths due to lung infections the changes of growth of the $2^1$- and $3^1$-groups were difficult to assess, but the animals were in poorer condition and failed to maintain their initial rate of growth.

These results harmonise with those reviewed earlier (Crabtree, 1949) where several workers found that a methyl group in the $4^1$-position of 4-dimethylaminoazobenzene or 4-methylaminoazobenzene caused an enormous fall in their carcinogenic potency. These Me-substituted carcinogens, like the $4^1$-compound used in the present experiments, are also capable of yielding p-AB by reductive fission and oxidation and this common biochemical feature may well be the cause of their similar biological behaviour.

### 2. *BY in Conjunction with Precursors of p-AB.*

In the previous section experiments have been described which show that a modification of the molecule of the carcinogen BY by the introduction of a $4^1$-methyl or $4^1$-carboxy group produces molecules with little or no carcinogenic power and this change in biological properties has been attributed hypothetically to a release of p-AB on metabolic breakdown.

The possibility of antagonising the potency of BY by the simultaneous feeding of other azo-compounds capable of liberating p-AB in the liver—the site of metabolic attack on azo-compounds—was then investigated. The azo-compounds chosen for this purpose were not only precursors of p-AB, but were themselves non-carcinogenic. The latter property, initially presumed on the basis of existing data, was later demonstrated experimentally. The five azo-compounds used—P1, P2, P3, P4, and P5—had, with the exception of P5, the simplest possible structures yielding innocuous breakdown products, thus avoiding, as far as possible, complicating factors which might lead to uncertainty in the interpretation of results. Two of them, P2 and P3, proved to be relatively more toxic than the others, possibly because their polar groups facilitated attachment to proteins and interference with normal enzyme function.

(a) *Effect of P1, P2, P3, P4, and P5 on the growth of rats.*

Compound P4, known to be non-carcinogenic, had been shown to enhance the growth-rate of rats when included in the standard semi-synthetic diet and this property was attributed to the liberation of p-AB by metabolic processes (Crabtree, 1949). Since all these compounds possess this potentiality, their effect on growth, when included in the basal diet at a concentration of 0·04 per cent, was investigated. The results are shown in Fig. 1.

Rats grew more rapidly when consuming any one of these substances, but not all to the same extent. There was no clear correlation between the degree of growth-stimulation produced by these compounds and their rate of metabolic breakdown or their solubility in fat-solvents. No doubt many factors contribute towards their nett effects on growth, but the outstanding common property is that of acting as precursors of p-AB.

FIG. 1.—Growth curves of rats fed on the basal diet alone (control), or with the addition of 0·04 per cent of one of the five azo-compounds (P1, P2, P3, P4, P5) which yield p-aminobenzoic acid on metabolic breakdown.

(b) *Effect of P1, P2, P3, P4, and P5, on the growth of rats consuming BY.*

In studying the possible anti-carcinogenic power of these azo-compounds it became evident that their addition to a BY-containing diet influenced the rate of growth and the general condition of the rats.

The basal diet alone permitted a slow increase of weight, but when supplemented by 0·04 per cent of BY a gradual fall in weight occurred and continued until the emergence of liver tumours caused death at an average time of 186 days.

By contrast, diets containing BY together with 0·04 per cent of any one of the substances P1, P2, P3, P4, or P5 promoted the growth of the rats in variable degree, but in all cases the growth-depressing effect of BY was antagonised. Three groups of rats, P1 + BY, P2 + BY, and P4 + BY suffered early losses from lung infections, but the remaining animals continued to grow steadily, as is shown in Fig. 2.

(c) *Effects of P1, P2, P3, P4 and P5 on the induction of liver tumours by BY.*

Six groups of 16 rats were fed on the basic diet containing 0·04 per cent BY and in five groups a further supplement of 0·04 per cent of P1, P2, P3, P4 or P5

FIG. 2.—Growth curves of rats fed on the basal diet. (*a*) Alone (control). (*b*) Containing 0·04 per cent BY. (*c*) Containing 0·04 per cent BY and 0·04 per cent of one of the five azo-compounds (P1, P2, P3, P4, P5) which yield p-aminobenzoic acid on metabolic breakdown.

FIG. 3.—Showing the retarding effect of azo-compounds which yield p-aminobenzoic acid on metabolic breakdown (P1, P2, P3, P4, P5) on the rate of induction of liver tumours by butter yellow (BY) fed for 350 days.

was incorporated into the diet. The groups are referred to as BY, P1 + BY, P2 + BY, P3 + BY, P4 + BY and P5 + BY, three of which (Fig. 3) were reduced in numbers by infections at an early stage.

The emergence of liver tumours was easily detectable by palpation. From the size of a pea the growth of an out-growing nodule could be followed over 3–4 weeks, when a 20–30 g. tumour signalled the approaching death of the rat.

The time of occurrence of these small nodules is recorded in Fig. 3, each rat receiving a single mark. Early stages of malignant transformation were therefore not assessed, but since the process of nodule formation with subsequent enlargment was a common factor in all rats, the marks give an approximate measure of the rate of incidence of tumours.

After 200 days, all rats consuming BY alone carried tumours, some large and others just palpable, and were killed or died at an average time of 186 days. At this time no palpable tumour was found in any of the five remaining groups, and they were maintained until the 350th day on their respective diets, except that during this latter period they were twice given ordinary laboratory food for one to two weeks to maintain health and thus possibly prolong the inhibitory action already demonstrated. Some tumours emerged during this period, and after 350 days, the BY-feeding was discontinued, but the precursors of p-AB were still fed at the reduced rate of 0·02 per cent, until the 570th day, when all surviving animals were killed. Twenty-five rats, out of an original sixty-two, were still free from palpable tumours, though the livers of most of these animals were not normal. The detailed results are gathered together in Fig. 3. It is notable that the survival time of some of these rats was longer than that of animals consuming the basal diet alone, suggesting that the deficiencies of this diet are partially compensated by the azo-compounds which function as precursors for p-AB.

### 3. *Experiment with O-aminoazotoluene.*

The experimental procedures were similar to those described for BY except that 0·04 per cent o-aminoazotoluene (O : O) was the carcinogen used. During the first year the average weight of rats consuming O : O alone increased by 25 per cent, while an increase of 34–46 per cent occurred in rats consuming the carcinogen supplemented by 0·04 per cent of P1, P2, P3, or P4.

From this time onwards the general health of some of the animals tended to deteriorate slowly, and in some cases slight weight losses were observed. In order to promote longer survival all groups of rats were fed on ordinary laboratory diet for two spells of fourteen days during the second year of the experiments. Each rat was killed when a liver tumour was palpable or when signs of ill-health were evident, and all survivors were killed after 715 days of treatment.

A summary of results is shown in Table III. The long period necessary for the induction of liver-tumours by O : O represents a large fraction of the life-span of rats, and this increases the difficulty of demonstrating any inhibitory effects which may result from the feeding of additional non-carcinogenic azo-compounds. The average time of survival, though somewhat longer in the groups consuming supplements of P1, P2, P3, or P4, does not adequately reflect the degree of inhibition of the rate of tumour induction. For this reason the results are classified under the three headings shown in Table III. When no gross tumour was apparent and no minute tumour foci were visible, the liver was described as

" normal " regardless of the well-known cellular changes which precede the emergence of tumours.

TABLE III.—*Induction of Liver Tumours by O-Aminoazotoluene (O : O) Fed in Conjunction with P1, P2, P3, or P4.*

| Azo-compounds. | Visible tumours. | Minute tumour foci. | " Normal " liver. | Days of treatment until death. Range (days). | Average (days). |
|---|---|---|---|---|---|
| O : O | 12 | 2 | 2 | 522–670 | 600 |
| O : O + P1 | 4 | 5 | 7 | 562–715 | 643 |
| O : O + P2 | 2 | 4 | 10 | 531–715 | 626 |
| O : O + P3 | 2 | 6 | 8 | 560–715 | 646 |
| O : O + P4 | 5 | 5 | 6 | 529–715 | 637 |

*Note on Method of Estimating the Times of Induction of Tumours.*

The criteria used by different workers for comparing the carcinogenic activity of substances which induce liver tumours have varied. In many cases records seem to refer to the time of emergence of large tumours and in other cases the effects were assessed by killing all the animals at some arbitrary time. Laparotomies performed before tumours were palpable have sometimes provided additional information. Unless the differences between the experimental groups are wide, the interpretation of results may still be equivocal. Perhaps an experiment in which large numbers of animals were used and random samples killed at short intervals would be the ideal.

The criterion used in this work has been the time of emergence of small palpable tumour nodules. Though all early changes are thus ignored, this baseline seems justifiable on the grounds that it is a common event in almost all rats, and that the subsequent growth of the tumour is rapid and, in a short time, lethal. In any case, the general condition of the animals provides a fair indication of differential effects and the terminal stages of loss of weight and tumour enlargement occupy a relatively small part of the experimental period.

With these reservations in mind the conclusion seems justified that inhibitions in the rate of tumour induction by BY and O : O are a sequel of the additional feeding of precursors of p-AB.

### SUMMARY.

1. Five azo-compounds,—P1, P2, P3, P4, and P5—which are potential precursors of p-aminobenzoic acid have been tested for anticarcinogenic action on 4-dimethyl-aminoazobenzene (BY). Their preparation and fate in the rat are described.

2. Rats consuming a basic diet containing 0·04 per cent of any one of these azo-compounds grew more quickly than rats fed on the basic diet alone. This augmentation of growth rate varied in degree with the compound used.

3. Rats on the same basic diet containing 0·04 per cent of BY progressively lost weight and liver tumours developed in all of them over a period of 200 days.

4. Rats consuming the BY-containing diet further supplemented with 0·04 per cent of any one of the compounds P1, P2, P3, P4, or P5 increased in weight, and the rate of incidence of liver tumours was retarded in all cases to a variable degree.

5. In parallel experiments in which o-aminoazotoluene was the carcinogen used, a similar lowered incidence of liver tumours was demonstrated.

I am indebted to the late Dr. E. Vazquez-Lopez for generous collaboration in this and other work.

### REFERENCES.

Bratton, A. C. and Marshall, E. K. Jr.—(1939) *J. biol. Chem.*, **128**, 537.
Bray, H. G., Neale, F. C. and Thorpe, W. V.—(1946) *Biochem. J.*, **40**, 134.
*Idem* and Thorpe, W. V.—(1948) *Ibid.*, **43**, 211.
*Idem*, Lake, H. J., Neale, F. C., Thorpe, W. V. and Wood, P. B.—(1948) *Ibid.*, **42**, 434.
Crabtree, H. G.—(1949) *Brit. J. Cancer*, **3**, 387.
Folin, O.—(1905–6) *J. biol. Chem.*, **1**, 131.
Kinosita, R.—(1936) *Gann*, **30**, 423.—(1937) *Trans. Jap. path. Soc.*, **27**, 665.
Perkin, W. H.—(1880) *J. chem. Soc.*, **37**, 554.
Peters, J. P. and van Slyke, D. D.—(1932) 'Quantitative Clinical Chemistry', Vol. II, p. 449. London (Bailliere Tindall & Cox).